# Traumatic oculomotor nerve palsy treated with transnasal endoscopic decompression through the optic strut

**DOI:** 10.3389/fsurg.2022.1051354

**Published:** 2023-01-06

**Authors:** Anqi Wang, Mian Wang, Yanqiao Wu, Yifan Zhao, Zhimin Wang, Likui Shen

**Affiliations:** ^1^ Department of Neurosurgery, Suzhou Kowloon Hospital, Shanghai Jiaotong University School of Medicine, Suzhou, China; ^2^ Department of Otorhinolaryngologic, The Affiliated Beijing Tsinghua Changgung Hospital of Tsinghua University, Beijing, China; ^3^ Department of Neurosurgery, Affiliated Dushu Lake Hospital of Soochow University, Suzhou, China

**Keywords:** traumatic oculomotor nerve palsy, optic strut triangle, superior orbital fissure, endoscopy, optic canal, decompression

## Abstract

**Objective:**

To present a surgical treatment regimen of transnasal endoscopic decompression through the optic strut for traumatic oculomotor nerve palsy based on the anatomical study of the superior orbital fissure and the oculomotor nerve fissure segment.

**Methods:**

The bone structure of the oculomotor nerve fissure segment and the important bone anatomical landmarks of the lateral wall of the sphenoidal sinus were identified on a dried skull and a cadaveric head, respectively, using a nasal endoscope, and a surgical plan was determined. Decompression was conducted on the orbital apex, the superior orbital fissure, the optic canal and the optic strut of the two patients in sequence, after which the degree and range of decompression were identified by three-dimensional (3D) computed tomography (CT).

**Results:**

The oculomotor nerve had a close correlation with the lateral surface of the optic strut. The transnasal endoscope was employed to identify the lateral optic-carotid recess (LOCR), as well as the positions of the optic nerve, internal carotid artery (ICA), and superior orbital fissure, collectively called the “optic strut triangle”. The surgical plans for decompression of the orbital apex, superior orbital fissure, optic canal, and optic strut and the necessity of optic strut drilling were determined, and the surgical procedures for safe drilling of the optic strut were elaborated. After surgery, the two patients had significantly improved symptoms, without complications. In addition, their postoperative CT showed that the medial margin of the superior orbital fissure was fully decompressed.

**Conclusion:**

The optic strut triangle is a crucial anatomical landmark in the decompression of the oculomotor nerve, and optic strut drilling is necessary for such decompression. For patients with traumatic oculomotor nerve palsy and fractures of the medial wall of the superior orbital fissure on CT, the oculomotor nerve fissure segment can be decompressed in an effective, complete and safe manner as per the surgical plan of decompressing the orbital apex, superior orbital fissure, optic canal and optic strut in turn under a transnasal endoscope, conducive to the recovery of neurological function of patients.

## Introduction

Oculomotor nerve palsy manifests itself through a number of symptoms such as ptosis, restricted upward, inward and downward movement of the eyeball, mydriasis, and lack of light reflex of the affected eye, and possibly diplopia. Unilateral isolated oculomotor nerve palsy is usually caused from vascular injuries in the subarachnoid space or cavernous sinus, as well as the microvascular infarction of the brainstem. Rarely, it can be a result of tumors, inflammation, vasculitis, infection, intracranial aneurysm, and traumas (1). In the neurosurgical disease spectrum, some carotid-posterior communicating aneurysms can contribute to unilateral oculomotor nerve palsy, and such compressive lesions are often accompanied by mydriasis (2). In primary craniocerebral traumas, symptoms of unilateral oculomotor nerve palsy are rare (3). Currently, traumatic oculomotor nerve palsy is mainly treated *via* steroid hormones, eye movement rehabilitation and botulinum toxin treatment, lacking an established treatment plan and standardized surgical method (4, 5). This lack of standarised treatment can lead to a long recovery time, with often uncertain outcome, failing to completely cure the disease (6). The symptoms of ptosis and diplopia affect the daily life of the patient, and some require reoperation by an ophthalmologist or a plastic surgeon. However, surgery for ptosis easily leads to over-correction or under-correction, and is extremely prone to exposure keratitis. The surgery for strabismus is able to correct the deviation of the eyeballs, but unable to restore eyeball movement (7, 8). Recently, it has been reported that early surgical decompression of the superior orbital fissure is recommended for patients with traumatic oculomotor nerve palsy and sphenoid fractures. Chen et al*.* conducted surgical decompression of the superior orbital fissure in six patients, achieving desirable outcomes (9). Lin et al*.* (5) made a retrospective analysis on 26 patients with traumatic oculomotor nerve palsy, and performed decompression of the superior orbital fissure in 6 patients with the superior orbital fissure compressed by sphenoid fracture fragments. They discovered that all patients who have underwent surgery recovered from the ptosis and external ophthalmoparesis. Furthermore, 4 patients with internal ophthalmoplegia also recovered at the same time. Liu et al*.* applied a combined decompression of the optic canal, superior orbital fissure and orbital apex with a transnasal endoscope in patients with superior orbital fissure syndrome (SOFS) with success (10). Up to now, transnasal endoscopic decompression in patients with traumatic oculomotor nerve palsy has been rarely reported.

In this study, the anatomical characteristics of the oculomotor nerve in the superior orbital fissure are presented under a transnasal endoscope, and a surgical plan for transnasal endoscopic decompression of the orbital apex, superior orbital fissure, optic canal and optic strut is proposed. In addition, the therapeutic effects of the procedure were assessed in 2 patients.

## Materials and methods

The bone anatomical structure of the superior orbital fissure on an adult dried skull was observed transnasally and orbitally using a transnasal endoscope. In addition, anatomical landmarks related to the lateral wall of the sphenoid sinus on an adult dual-color latex-infused cadaveric head were observed by the transnasal endoscope. Before surgery, the surgical indications of two patients with traumatic oculomotor nerve palsy and superior orbital fissure fracture were assessed through computed tomography (CT) scan and three-dimensional (3D) reconstruction. Subsequently, the patients underwent transnasal endoscopic decompression of the oculomotor nerve fissure segment. The outcome of the surgery was evaluated based on postoperative CT scan (3D reconstruction) and clinical symptoms. A Storz endoscope (0°, 4 mm) was employed for dissection and surgical video recording. The anatomy and cases described in the research were used mononostril approach.All studies on humans were approved by the Ethics Committee of Suzhou Kowloon Hospital Shanghai Jiao Tong University School of Medicine and informed consent was obtained from patients for the use of their imaging data and facial signs.

### Anatomical landmarks of the superior orbital fissure and the lateral wall of the sphenoid sinus

**Figure 1:**
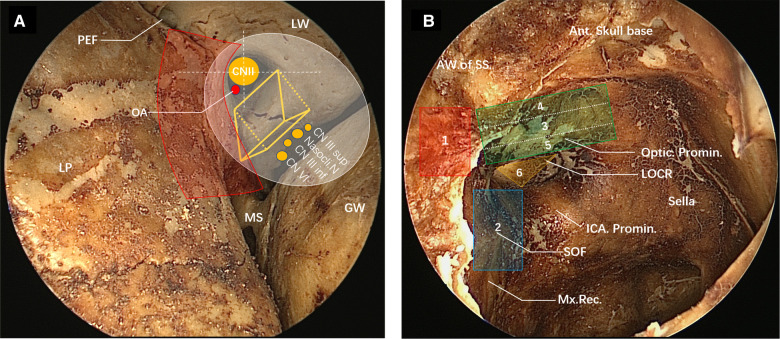
View of the superior orbital fissure and lateral wall of the sphenoid sinus on the dried skull under the nasal endoscope. (**A**) Transorbital view of the partial superior orbital fissure (left). The medial margin of superior orbital fracture consists, from top to bottom, of the lateral surface of the optic strut (optic strut fractures shown in the figure), part of the body of the sphenoid bone and the maxillary strut. The lateral margin of the superior orbital fissure consists of the great wing of the sphenoid bone. The optic strut can be seen as a triangular prism structure (yellow triangular prism structure in the figure), with its superior bottom connected to the small wing of the sphenoid bone and its inferior bottom connected to the body of the sphenoid bone, jointly forming an optic canal, through which the optic nerve and the ophthalmic artery pass. The optic canal is divided into four quadrants by white dotted lines, and the ophthalmic artery enters the orbit from the right lower quadrant. The lateral wall of the optic strut is adjacent to the nerves emerging from the cavernous sinus, namely the superior branch of the oculomotor nerve, the nasociliary nerve, the inferior branch of the oculomotor nerve and the abducent nerve from top to bottom, and they enter the orbit through the common tendinous ring (white area). The red area is the range of decompression of the optic canal, the superior orbital fissure and the orbital apex. Posterior ethmoidal foramen can be seen on the medial wall of the roof of the orbit, and the medial wall of the orbit is the lamina papyracea. (**B**) View of the lateral wall of the sphenoid sinus under the nasal endoscope (right). The yellow triangle area is the inferior bottom of the optic strut in a triangular prism structure (LOCR). The optic nerve prominence, the superior orbital fissure (blue area) and the ICA prominence form the three sides of the optic strut triangle. The red area is the approximate range of decompression of the orbital apex. The superior orbital fissure (overall blue area) is adjacent inferiorly to the maxillary strut depression, and superiorly to the optic strut. The residual anterior wall of the sphenoid sinus following sphenoidostomy is shown on the left side of the view. The order of the decompression of the oculomotor nerve fissure segment is as follows: (1) orbital apex, (2) superior orbital fissure, (3) inferomedial wall of the optic canal, (4) superior wall of the optic canal, (5) inferior wall of the optic canal, and (6) optic strut.

The superior orbital fissure is a vital space connecting the middle cranial fossa and the orbit, and it is located between the great wing, small wing and body of sphenoid bone, shaped like a triangle. The inner side of the superior orbital fissure forms the base of the wide triangle, and the outer side is a narrow tip formed between the great wing and the small wing of the sphenoid bone. The superior orbital fissure is not located on the standard coronal plane; its lateral tip points to the anterolateral side and its medial margin consists of the lateral margin of the optic strut and partial body of sphenoid bone (11, 12). Observed from an orbital perspective, the medial surface of the superior orbital fissure is composed of three parts: the superior, the median and the inferior part, corresponding to the lateral margin of the optic strut, the lateral surface of the sphenoid bone, and the maxillary strut (Figure 1A). The optic strut is a cylindrical structure, but it can also be seen as a triangular prism from its adjoining structure (Figure 1A). Its medial surface forms the lateral wall of the optic canal, close to the optic nerve, and the posterior wall clings to the anterior curvature of the cavernous sinus segment of the internal carotid artery (ICA). Its lateral wall comprises the middle of the medial margin of the superior orbital fissure, and triangles on the superior and inferior bottoms separately connected the small wing of sphenoid bone and originated from the body of sphenoid bone.

The common origin of the four rectus oculi is the tendinous ring (white oval in Figure 1A), which surrounds the optic canal and the center of the superior orbital fissure, dividing in this way the superior orbital fissure into three parts: a superolateral, a central, and an inferomedial (13). The common tendinous ring surrounds the optic neuropore, oculomotor neuropore and the central part of the superior orbital fissure (13, 14). The superior margin of the oculomotor neuropore consists of the annular tendon and part of the small wing of the sphenoid bone. The lateral margin refers to the annular tendon and its protrusion, attaching to the lateral margin of the superior orbital fissure. The medial margin consists of the lateral margin of the optic strut and part of the body of the sphenoid bone. Laconetta et al*.* divided the oculomotor nerve into five segments by its running: cisternal segment, petrosal ligament segment, cavernous sinus segment, superior orbital fissure segment, and orbital segment (15). The oculomotor nerve fissure segment starts from the oculomotor nerve exiting the anterior wall of the cavernous sinus, passes through the superior orbital fissure, and enters the orbit through the common tendinous ring. The oculomotor nerve separates into superior and inferior branches 2–3 mm before entering the superior orbital fissure, and the superior and inferior branches pass through the central part (oculomotor neuropore) of the superior orbital fissure and enter the orbit closely along the lateral margin of the optic strut (13, 15). The nasociliary nerve and the abducent nerve enter the common tendinous ring along with the oculomotor nerve (Figure 1A). Adequate decompression of the orbital apex and the superior orbital fissure by the transnasal endoscope is required for decompression of the common tendinous ring. The decompression range of the orbital apex and superior orbital fissure are denoted in red. Removing the bone in this area enabled the decompression of the medial margin of the common tendinous ring. After optic strut drilling, the oculomotor nerve at the medial margin of the fissure segment was completely decompressed, and the optic canal, except for the bone in the superior right quadrant, was ground and decompressed to a 270° decompression range.

**Figure 2:**
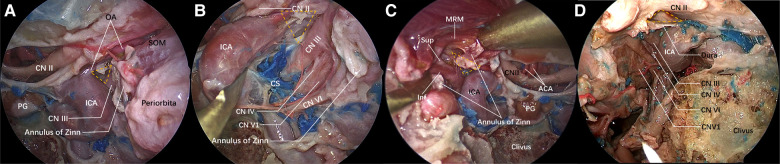
Structure adjacent to the “optic strut triangle” under the nasal endoscope. The lateral wall of the sphenoid sinus and the orbital apex bone are ground. The yellow dotted triangle is the ground optic strut triangle. (**A**) View of the lateral wall of the left sphenoid sinus under the nasal endoscope. The optic nerve sheath is incised, and the ophthalmic artery emerges from the ICA horizontal segment and enters the orbit below the optic nerve along the upper lateral surface of the optic strut. The other two surfaces of the optic strut are adjacent to the ICA and the oculomotor nerve, respectively. A part of the exposed oculomotor nerve is shown in the figure, the space orbit apex is the incised common tendinous ring and orbital periosteum, and the superior oblique muscle is exposed. (**B**) The ICA is displaced medially to expose part of the oculomotor nerve. The nerves emerging from the medial wall of the cavernous sinus are oculomotor nerve, trochlear nerve, ophthalmic nerve and abducent nerve from top to bottom. The oculomotor nerve is closely connected with the optic strut. (**C**) On the other side of the same specimen, the medial rectus muscle is lifted to expose the superior and inferior branches of the oculomotor nerve. (**D**) The optic strut is ground while the dura mater around the optic strut is preserved. The oculomotor nerve, abducent nerve, and trochlear nerve and ophthalmic nerve emerging from the lateral wall of the cavernous sinus are identified.

When the lateral wall bone of the sphenoid sinus is observed under the transnasal endoscope, the inferior bottom of the optic strut can be seen, in the case of good pneumatization of the sphenoid sinus, forming an lateral optic-carotid recess (LOCR). The “triangle” on the inferior bottom was contiguous to the ICA, optic nerve, and superior orbital fissure, respectively (Figure 1B). The superior and inferior branches of the oculomotor nerve cling to the lateral surface of the optic strut and enter the orbit through the body of the sphenoid bone on the medial wall of the superior orbital fissure (Figure 2A).

In conclusion, the oculomotor nerve can be easily compressed by dislocated fracture fragments, result of alterations of the bone structure of the medial margin of the oculomotor neuropore due to trauma and can cause nerve damage, edema and oculomotor nerve palsy symptoms. For this reason, decompression of the bone at the medial margin of the oculomotor nerve fissure segment, also called the orbital apex, part of the body of the sphenoid bone (the orbital apex and superior orbital fissure as seen under the transnasal endoscope), and the optic strut, is necessary.

### Spatial relationship between the oculomotor nerve fissure segment and the optic strut under the transnasal endoscope

Identifying important anatomical landmarks under the transnasal endoscope is very important. Wrong identification can lead to the damage of important blood vessels and nerves and result in severe complications. Alfieri et al*.* explained the importance of the “optic strut triangle” in detail from the perspective of a transnasal endoscope (16). In this study, the optic strut triangle was used as a vital anatomical landmark (yellow dotted triangle in Figure 2), based on which the ICA, the optic nerve and the superior orbital fissure were identified. The lateral wall bone of the sphenoid sinus was ground to expose the oculomotor nerve fissure segment and its surrounding important anatomical structures. Next, a safe and effective surgical plan was formulated following a detailed understanding of the surrounding anatomical structures, and the way to grind the optic strut safely and effectively was explored, in order to completely decompress the oculomotor nerve fissure segment.

The yellow dotted triangle denotes the ground bottom wall of the optic strut. The superior margin was the optic nerve, exposed after the dura mater was cut open. The ophthalmic artery originating from the ICA, ran below the optic nerve and entered the orbit just along the superior margin of the optic strut triangle. The common tendinous ring was dissected to expose the oculomotor nerve fissure segment and the medial orbital segment, with the former attached to the outer side of the optic strut triangle (Figure 2A). Then, the ICA was displaced medially to expose the nerves running through the lateral wall of the cavernous sinus: the oculomotor nerve, the trochlear nerve, the ophthalmic nerve, and the abducent nerve, running inside the cavernous sinus from top to bottom (Figure 2B). On the right side of the same specimen, the superior and inferior branches of the oculomotor nerve were exposed after dissecting the common tendinous ring and lifting the medial rectus muscle, as they clung to the optic strut (Figure 2C). With respect to the other specimen (Figure 2D), the bone of the orbital apex and superior orbital fissure was successively ground, and the medial wall, top wall, and inferior wall bone of the optic canal was then sequentially ground to achieve a 180° exposure of the optic nerve. Prior to optic strut drilling, decompression was preferentially performed on the bone on the surface of the surrounding important nerves, reducing the compression of the cranial nerve (CN) II and III during the resection of the optic strut and avoiding the occurrence of complications. During optic strut drilling, it was found that decompression in the order shown in the figure, minimized the compression and injury of nerves, and reduced the compression and injury due to the instruments used in the surgery and the bone around the nerves. After completing the optic strut drilling, a 270° decompression of the optic nerve was achieved, and the bone at the medial margin of the oculomotor nerve fissure segment was completely opened (Figures 2A–D).

### Transnasal endoscopic decompression of oculomotor nerve through the optic strut

Case 1: A 36-year-old man complained of blurred vision in the left eye for 1 h following head trauma and was admitted to the hospital. After admission, the neurological examination showed that the pupil of the left eye was 4 mm, and lacked direct and indirect light reflex. In addition, the patient complained that the left eye had no light perception. Moreover, ptosis, and limited upward, inward and downward movement of the left eyeball were observed. Based on the above observations, an optic nerve and oculomotor nerve injury of the left eye was considered. Cranial CT and 3D reconstruction were carried out, and the results revealed multiple fractures of the left frontotemporal parietal bone, great wing of sphenoid bone, and lateral wall of the left orbit, fracture of the plateau of left sphenoid bone, and involvement of the left optic canal. Furthermore, CT 3D reconstruction displayed fracture lines around the superior orbital fissure from different angles (Figures 5A–C). Additionally, a small amount of epidural hematoma was observed in the left temporal region from the brain window. It was confirmed that the surrounding bone of the optic canal and superior orbital fissure was fractured, with symptoms of neurological deficit, and the epidural hematoma had no progression. It was decided to perform transnasal endoscopic decompression. The surgical decompression was conducted in the order shown in Figure 1B. The 270° decompression of the optic nerve was achieved while decompressing the oculomotor nerve fissure segment.

**Figure 3:**
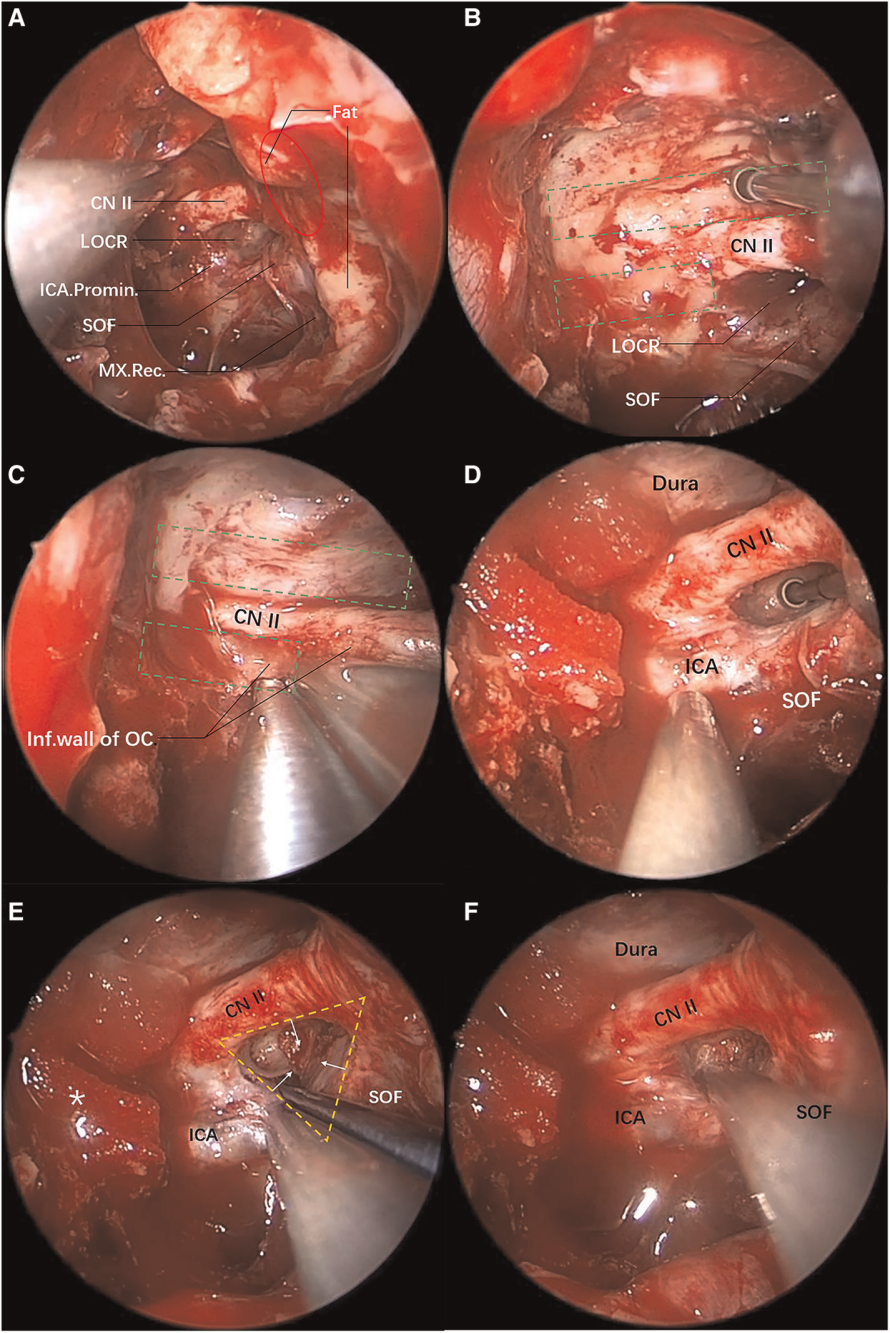
Transnasal endoscopic decompression of oculomotor nerve through the optic strut. The yellow dotted triangle area denotes the range of the optic strut triangle, and the red oval area the range of the orbital apex. (**A**) View of the lateral wall of the left sphenoid sinus after the anterior wall of the sphenoid sinus is opened under the transnasal endoscope. The sphenoid sinus pneumatization is good on the left side, LOCR is an obvious depression and its three sides are the ICA, the superior orbital fissure and the optic nerve, respectively. The fat in the orbital apex is exposed due to traumatic fractures. There is a depression below the superior orbital fissure, namely a maxillary strut depression, which is the inferior margin of the superior orbital fissure. (**B**) The superior wall bone of the optic nerve is eggshelled and the anterior skull base dura mater is exposed. The green dotted rectangle (top) denotes the drilling range. (**C**) The inferior wall bone of the optic nerve is eggshelled. The inferior wall bone is removed with a gun-shaped rongeur. The green dotted rectangle (bottom) shows the drilling range. (**D**) The optic strut is eggshelled. (**E**) Three surfaces of the triangular prism-shaped optic strut are eggshelled. The eggshelled optic strut is peeled off using a detacher in the direction of the yellow arrow. The yellow dotted triangle is the optic strut triangle, and the asterisk on the left side indicates the gelatin sponge used for compression hemostasis of the superior intercavernous sinus. (**F**) Decompression is performed successively on the orbital apex, the superior orbital fissure, the optic canal and the optic strut.

Following general anesthesia, routine iodine sterilization was performed at the facial area, and a drape was placed exposing both nostrils. Subsequently, the nasal cavities were further flushed with iodophor, and the left nasal mucosa was infiltrated with 1:10,000 epinephrine cotton pads. The uncinate process and ethmoid vesicle were excised in turn, the superior meatus, the posterior ethmoid and the anterior wall of the sphenoid sinus were opened at the horizontal and vertical parts of the middle nasal concha plate, and the mucosa of the sphenoid sinus was radically resected, followed by the exposure of the bony landmarks. Firstly, the optic nerve prominence, the ICA prominence, and the superior orbital fissure were identified based on the optic strut triangle (LOCR). The position of the posterior ethmoidal artery canal (covered by the exposed intraorbital fat in the figure) and the position of the maxillary strut depression were determined, in order to identify the anterior-posterior range of the orbital apex and the inferior boundary of the superior orbital fissure (Figure 3A). Then, the orbital apex, the superior orbital fissure, and the medial inferior wall of the optic nerve were outlined using eggshell technique (in this case, the intraorbital fat was exposed due to the fracture of the superior orbital fissure and orbital apex). Subsequently, the superior wall of the optic nerve was outlined (Figure 3B), Then, the inferior wall bone of the optic nerve was ground, and the residual bone was removed with a gun-shaped rongeur (Figure 3C), reaching a decompression of roughly 180° of the optic canal, and the bone on the medial surface of the oculomotor nerve was removed. Based on the intrasurgical anatomical assessment, further optic strut drilling was decided for better decompression. The superior part of the optic strut triangle was the decompressed optic nerve, and the inferolateral part was the oculomotor nerve fissure segment running to the superior orbital fissure (Figure 3D). The optic strut bone was hollowed out from the center to the periphery, and the adhesion between the cortical bone of the strut and the dura mater was peeled off in the direction shown in Figure 3E. At this step, a 270° decompression of the optic canal was reached, and the complete decompression of the medial side of the oculomotor nerve fissure segment was achieved (optic strut and partial body of sphenoid bone in the superior orbital fissure) (Figure 3F). Lastly, wet compress with dexamethasone was performed locally, and the lateral wall of the nasal cavity was further filled with gelatin sponges to completely stop the bleeding of the nasal mucosa. After surgery, steroid hormones were administrated continuously to relieve nerve edema. Four hours following surgery, a thin-slice cranial CT was performed to clearly define the degree of decompression and changes in intracranial hemorrhage.

Case 2: A 46-year-old woman suffered a head injury due to accidental fall while riding a bicycle two months ago, with a transient coma. Cranial CT revealed a small amount of bleeding in the basal ganglia area. The patient had difficulty lifting the left eyelid, as well as mydriasis (but with good visual acuity), after the injury. She received steroids and rehabilitation therapies in another hospital. At day 60 after the injury, ptosis was observed in the left eye (Figure 8A). The left eye had a pupil of 5 mm, no direct and indirect light reflexes, and severe diplopia. The patient was troubled by appearance problems leading to the visit to our hospital. Orbital CT done at the initial hospital showed a fracture of the medial wall of the left superior orbital fissure after the injury. Cranial CT and 3D reconstruction were performed and revealed that the fracture site was basically healed. Following evaluation, it was decided to carry out transnasal endoscopic decompression of the oculomotor nerve through the optic strut.

**Figure 4:**
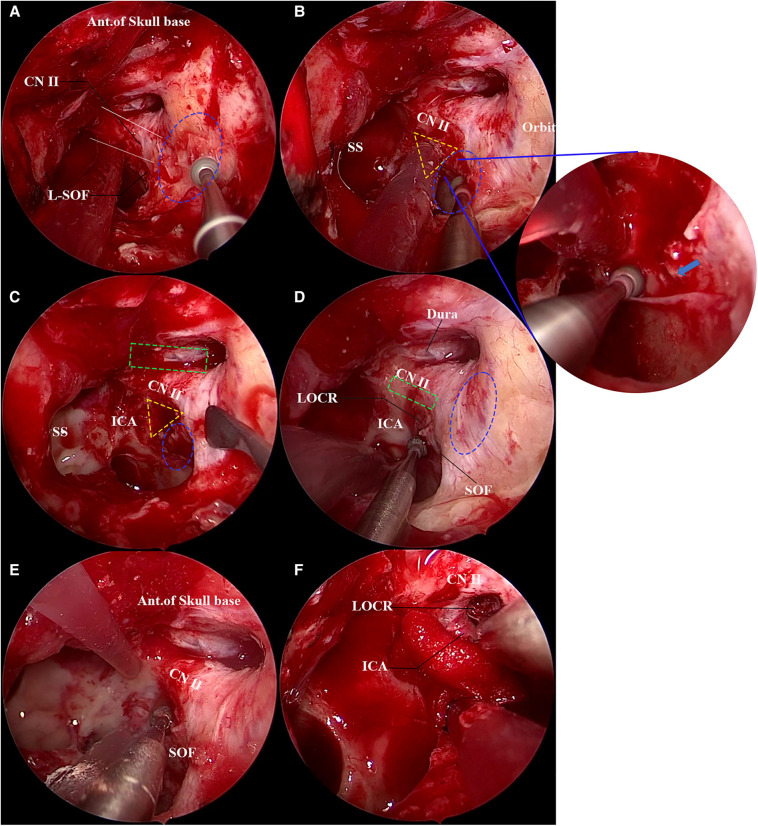
Transnasal endoscopic decompression of oculomotor nerve through the optic strut. The yellow dotted triangle area is the approximate range of the optic strut triangle. (**A**) The lateral wall of the left sphenoid sinus is exposed and the orbital apex decompression is performed. The orbital apex bone is being eggshelled using a high-speed burr in the figure. The sphenoid sinus pneumatization of this patient is poor, and the LOCR is not clearly displayed. The blue dotted oval area depicts the range of the orbital apex and the white solid line the range of the optic nerve, and the anterior skull base is in the upper field of view. (**B**) Nasal endoscopic decompression of superior orbital fissure. The blue dotted oval area shows the superior orbital fissure. The optic strut triangle can be observed, but its boundary is unclear. The superior orbital fissure bone is being eggshelled using a high-speed burr in the figure. The right image shows a magnification of the superior orbital fissure, with a fracture line (blue arrow) below the mucosa. (**C**) Decompression of optic canal. After decompression of the inferomedial and superior walls of the optic canal, the anterior skull base dura mater (green dotted rectangle) is exposed. The blue dotted oval area shows the range of the superior orbital fissure. (**D**) The boundary of the optic strut triangle is defined. The inferior wall of the optic nerve (green dotted rectangle) is eggshelled, and the bone around the optic strut triangle is ground to determine its boundary. The blue dotted oval area is the range of the orbital apex. (**E**) Decompression of the optic strut. The optic strut is decompressed under continuous water flow; it can be seen that a 180° decompression is achieved for the optic nerve, and both the superior orbital fissure and orbital apex are sufficiently decompressed. (**F**) The optic strut is eggshelled, the optic strut is removed, and the oculomotor nerve fissure segment is sufficiently decompressed.

Similar to the first case, the anterior wall of the left sphenoid sinus was opened through the middle nasal concha plate and the posterior ethmoid, and the bone landmarks were exposed after radical treatment of the sphenoid sinus mucosa. The “optic strut triangle” on the lateral wall of the sphenoid sinus was, then, identified. In this case, however, the optic strut triangle was difficult to identify due to poor pneumatization of the optic strut. Hence, the position of the orbital apex was firstly determined through the orbital cardboard for the decompression of the orbital apex (Figure 4A). After that, the optic nerve and superior orbital fissure could be clearly identified. Then, the outline of the optic strut triangle was identified, the superior orbital fissure was located and decompressed, and the fracture line of the superior orbital fissure was found (Figure 4B). Next, the inferomedial, superior and inferior walls of the optic nerve were outlined (Figures 4C,D), and the decompression of the orbital apex, the superior orbital fissure, the optic canal and the optic strut was conducted in sequence. In this case, the optic strut triangle had no obvious depression, with unclear boundaries. Therefore, the surrounding bone was outlined to identify the boundary of the optic strut triangle (Figure 4D) to avoid damage to surrounding blood vessels and nerves during the decompression of the optic strut. Following the identification of the boundary of the optic strut triangle, the optic strut bone was hollowed out. The hollowed optic strut and the eggshelled bone fragments were observed, and complete decompression of the inner side of the oculomotor nerve fissure segment was achieved (Figures 4E,F). After surgery, steroid hormones were given to relieve possible edema of nerves.

### Assessment of decompression degree and clinical outcomes

**Figure 5:**
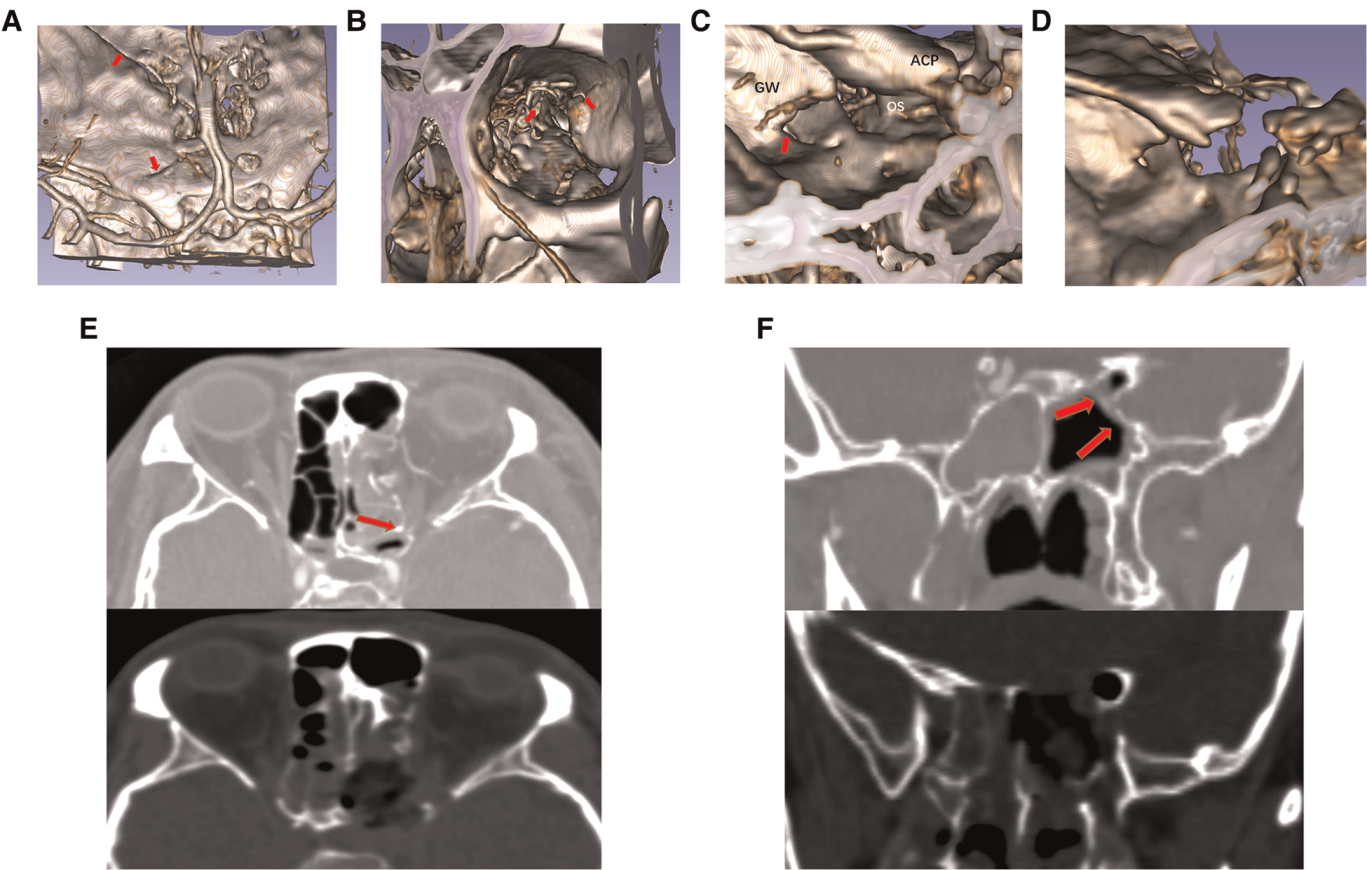
Comparison of preoperative and postoperative imaging data. 3D views of patients’ left orbit from different angles are shown in (**A–C**). The red arrow indicates the fracture line. (**A**) The fractures of the small wing of the sphenoid bone involve the superior wall of the optic nerve. (**B**) Fracture line of the superior orbital fissure from the orbital perspective. (**C**) Observation of the fracture line of the superior orbital fissure from the upper left of the skull base. (**D**) Thin-slice CT 3D reconstruction image after surgery. The preoperative (top) and postoperative (bottom) images of the patients are shown in E-F. The red arrow indicates the fracture line. (**E**) Comparison of preoperative and postoperative cranial axial CT findings. (**F**) Comparison of preoperative and postoperative coronal cranial CT findings. The upper red arrow indicates the optic canal fracture line, and the lower red arrow indicates the fracture line on the superior medial wall of the superior orbital fissure (corresponding to the oculomotor nerve fissure segment).

Case 1: The preoperative and postoperative thin-slice CT and 3D reconstruction images were compared, and it was found that the medial margin of superior orbital fissure and the optic canal were sufficiently decompressed, and the medial wall of the oculomotor nerve fissure segment (optic strut and part of body of sphenoid bone) were removed, including part of the anterior clinoid process (Figures 5A–D). Coronal CT showed the fracture line of the optic canal and the medial wall of the superior orbital fissure, and both the optic strut and the superior orbital fissure were sufficiently decompressed after surgery (Figures 5E,F). After surgery, the patient's visual acuity was gradually restored and the symptoms of oculomotor nerve palsy rapidly improved.

**Figure 6:**
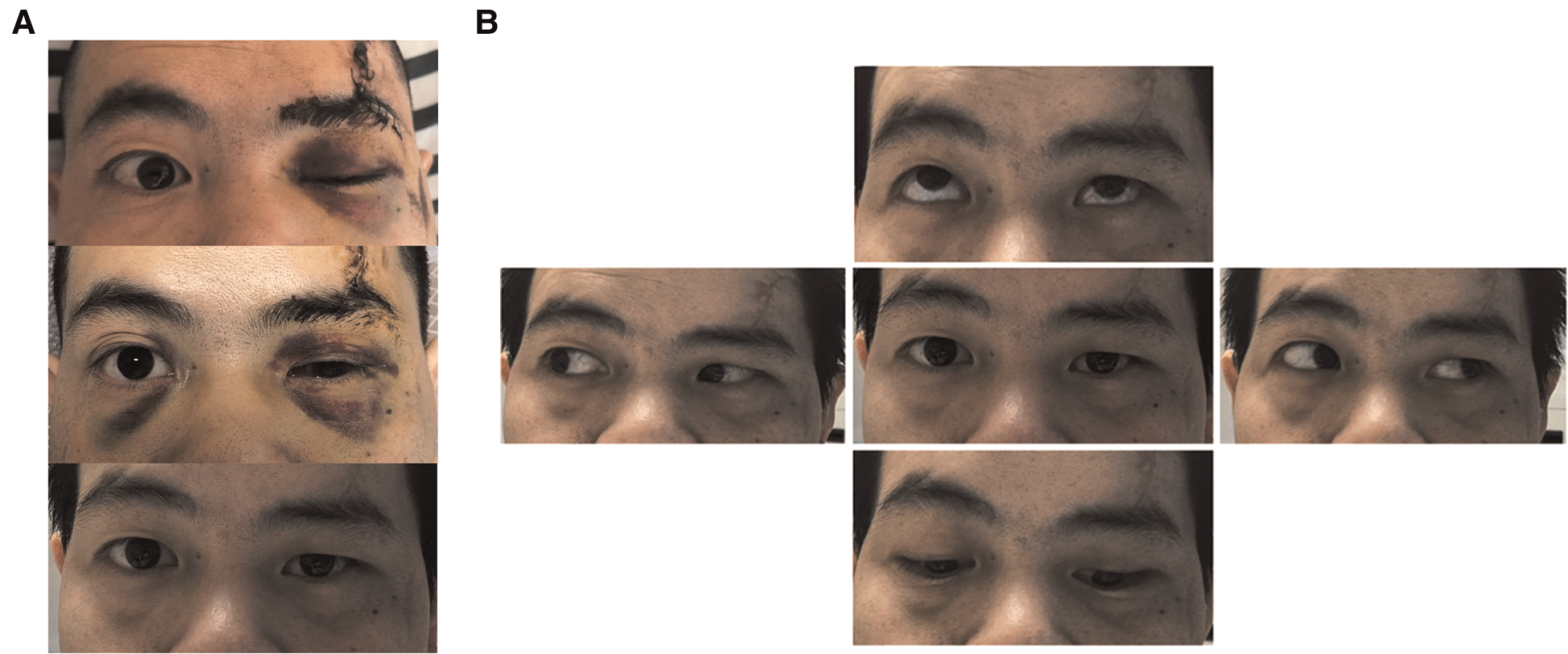
Changes in ptosis symptoms and eyeball movement 1 month after surgery. (**A**) Change in preoperative and postoperative ptosis symptoms: before surgery, 3 days after surgery and 1 month after surgery from top to bottom. (**B**) Upward, downward, leftward and rightward movement of the eyeball 1 month after surgery.

The postoperative conditions of ptosis and eyeball movement were recorded (Figures 6A,B), and our assessment showed a significant improvement in the ptosis symptoms within 3 days after surgery. At the 1-month follow-up, it was found that ptosis almost disappeared and eyeball movement was completely restored.

**Figure 7:**
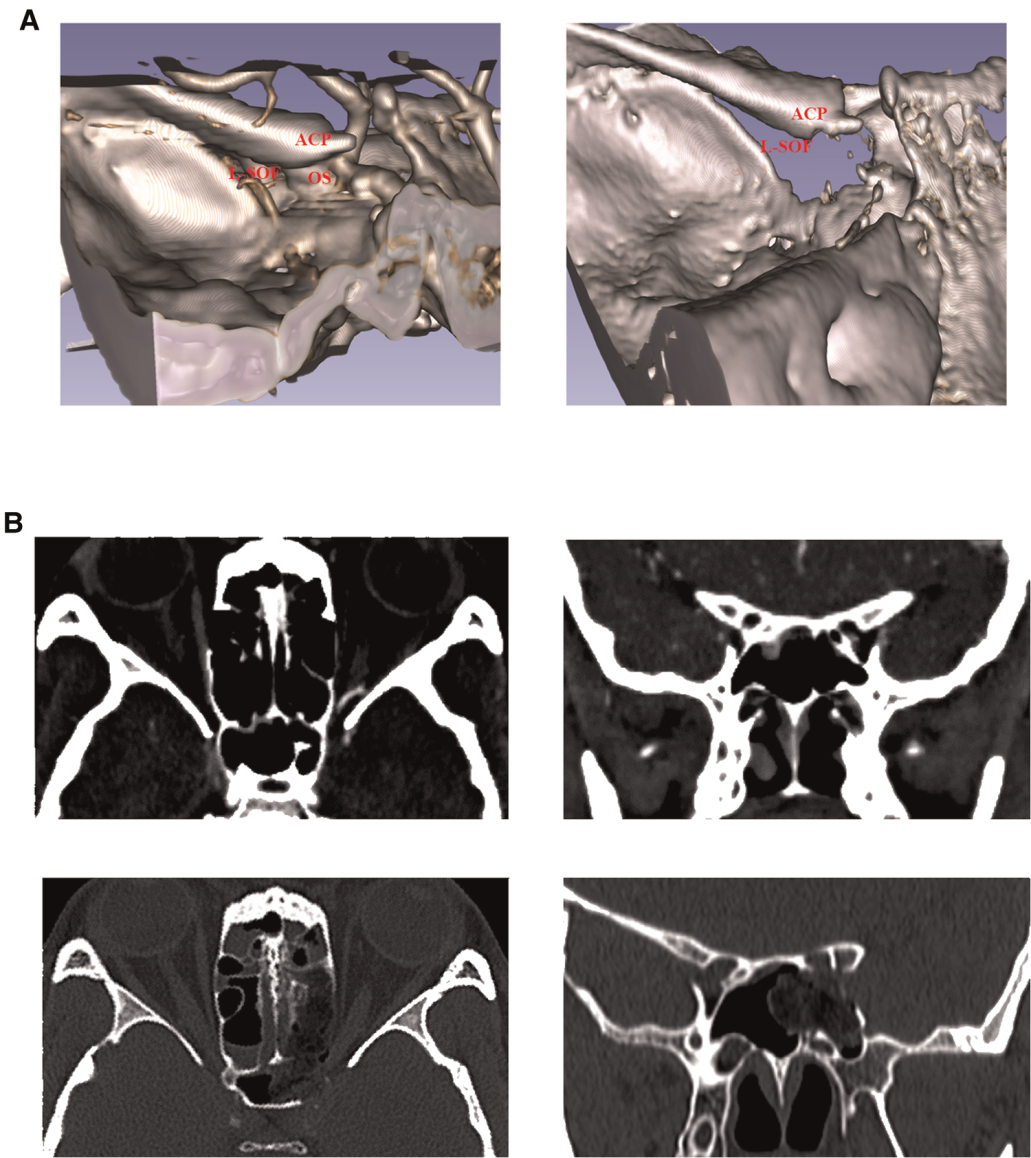
Comparison of preoperative and postoperative imaging data. (**A**) Observation of superior orbital fissure from the upper left of the skull base. Preoperative and postoperative 3D reconstruction images are shown on the left and right, respectively. It can be seen that the optic strut and part of the body of the sphenoid bone are resected. (**B**) Postoperative thin-slice CT reexamination. Left: Comparison of axial CT. Right: Comparison of coronal CT. Top: Preoperative images. Bottom: Postoperative images.

Case 2: Comparison of preoperative and postoperative 3D reconstruction images showed that the optic strut, the body of the sphenoid bone and part of the medial wall of the orbit were resected, and the medial margin of the superior orbital fissure was decompressed (Figure 7A). It could be seen from the comparison of preoperative and postoperative axial CT and coronal CT that the medial margin of the superior orbital fissure, including the optic strut, was resected (Figure 7B).

**Figure 8:**
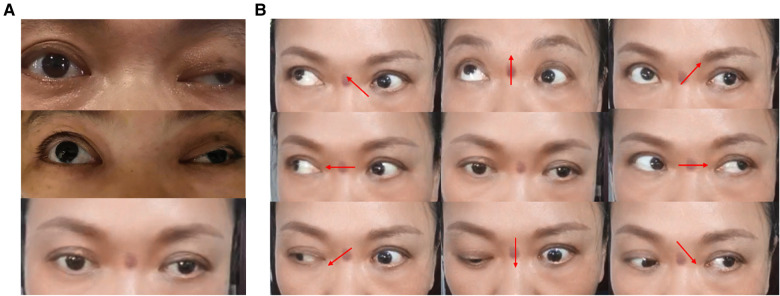
Changes in ptosis symptoms and eyeball movement 1 month after surgery. (**A**) Change in preoperative and postoperative ptosis symptoms: before surgery, 1 h after surgery and 1 month after surgery from top to bottom. (**B**) Changes in eyeball movement 1 month after surgery. Upward and downward movement disorder of the left eyeball remains.

Ptosis symptoms were assessed immediately 1 h after surgery. Comparison with preoperative conditions revealed that the black iris was more exposed when the eyes were opened, and ptosis gradually recovered during this period. One month later, ptosis symptoms in the left eye almost disappeared (Figure 8A), but the upward and downward movement disorder of the eyeball remained (Figure 8B).

## Discussion

Traumatic oculomotor nerve palsy generally occurs following craniocerebral traumas, including mild, moderate and severe craniocerebral traumas (17, 18). Moderate to severe craniocerebral traumas are often accompanied by multiple permanent neurological deficits (19). Uberti et al. suggested that traumatic oculomotor nerve injury may be attributed to the following four mechanisms: (1) The nerve root of the midbrain export is directly injured by a rotational shear force, (2) The oculomotor nerve is fixed to the petroclinoid ligament at the lower end of the oculomotor nerve triangle, so a nerve traction injury is directly caused due to the brain stem displacement after trauma, (3) Direct vascular compression is caused due to ICA dissection or pseudoaneurysm, (4) The blood supply to the oculomotor nerve is damaged (20).

The two cases of traumatic oculomotor nerve palsy reported here both suffered injuries in the forehead and around the orbit, and had fractures of the medial margin of the superior orbital fissure. One case had fractures of the lateral wall of the superior orbital fissure. The possible reason is that the stress applied to the lateral margin of the orbit is transmitted to the space orbit apex through the zygomatic and the sphenoid bone, resulting in fractures in weak areas such as the medial wall of the superior orbital fissure. Subsequently, the oculomotor nerve is compressed, causing dysfunction, which can also indirectly lead to nerve edema and inflammatory response. Therefore, steroid hormones are effective for post-traumatic SOFS and orbital apex syndrome (21). However, surgical decompression should be considered as early as possible for patients with definite fracture compression, especially those with no response to conservative treatment or gradual deterioration of neurological function. It is often believed that early surgical decompression will worsen tissue swelling and intraocular pressure, thus aggravating the CN palsy in the superior orbital fissure. It has been proved, however, that early surgical intervention has no negative impact on the recovery of CN (5, 9), possibly because the optic strut, orbital apex and superior orbital fissure are thoroughly decompressed, direct compression is relieved and aggravation of injury due to secondary edema is, therefore, prevented. However, high-dose steroid therapy is also required. In a study of optic-nerve injuries, patients treated with steroids therapy plus surgery had significantly better outcomes than those treated with steroids therapy alone (22). Whether this is the same as oculomotor nerve injury needs to be further studied, More cases and randomized controlled studies are needed for verification.At the same time, from the perspective of the topographic anatomy of the superior orbital fissure, the oculomotor nerve fissure segment is close to the optic strut and the body of the sphenoid bone on the medial wall, and enters the orbit (13, 15). The fracture of the medial wall of the superior orbital fissure is more likely to involve and injure the oculomotor nerve than the lateral wall. The optic strut is the bone applying the most direct compression when the oculomotor nerve enters the orbit through the cavernous sinus, in which the oculomotor nerve separates into superior and inferior branches. The superior branch anteriorly and superiorly enters the orbit through the common tendinous ring, so patients with traumatic oculomotor nerve palsy often present with superior branch damage. In the case of a definite superior orbital fissure fracture, surgical decompression is required for both medial and lateral fractures of the superior orbital fissure.

It is very important to identify the orbital apex, the superior orbital fissure and the optic strut under a transnasal endoscope. Wrong identification can lead to severe postoperative complications. Tepedino et al. used the ethmoid crest and posterior nasal arch as anatomical landmarks for locating the superior orbital fissure (23). Muller's muscle is considered a marker for identifying the optic strut, the maxillary strut and the superior orbital fissure, but it is rarely used clinically (24, 25). Liu et al. described a “π” structure for the identification of the optic canal, the superior orbital fissure and the orbital apex (10). Alfieri et al*.* explained in detail the composition of the “optic strut triangle” under the transnasal endoscope, which is essential in clinical work and this study. The optic strut is adjacent to three important structures, so it is regarded as a 3D structure in a triangular prism shape. However, it is worth noting that the “optic strut triangle” can only be clearly identified when the sphenoid sinus pneumatization is good, and an experienced skull base surgeon is required. In Case 2, the LOCR was almost undetectable, and the depression between the superior optic nerve and the anterior skull base was easily mistaken for the LOCR. If conducted in the above procedures, the surgery will be significantly less difficult. When the position of the LOCR cannot be identified, decompression of the orbital apex and superior orbital fissure should be given priority, and the position of the optic nerve and ICA can be determined through the orbital apex and superior orbital fissure, thus allowing the identification of the boundary of the optic strut triangle. To avoid damaging important nerves and blood vessels, neuronavigation is recommended when anatomical structures are not clearly identified.

It is necessary to first identify the location of the optic strut on the lateral wall of the sphenoid sinus and the important structures adjacent to it before being ground. Second, CN II and CN III will inevitably be squeezed or pushed when the eggshelled optic strut is ground and peeled off. To reduce nerve disturbance, priority should be given to decompression of the orbital apex and the superior orbital fissure. There are different definitions for the range of the orbital apex. Some scholars argue that the orbital apex does not include the superior orbital fissure and the optic canal (23, 25), while some believe that the orbital apex segment starts at the posterior ethmoidal canal to the optic canal (26). We think that the secondary edema following the local injury of the orbital apex will cause further damage to the high-density neurovascular structure in the common tendinous ring, so thorough decompression is required for the orbital apex and the superior orbital fissure, and the common tendinous ring can be incised for further decompression if necessary. As for whether it is needed to incise the common tendinous ring in oculomotor nerve decompression, we believe that as in optic nerve decompression, blindly incising the dura mater may cause unnecessary complications (27, 28). The decompression of the superior orbital fissure is bounded by the superior margin of the optic strut triangle and the maximal strut. It is unnecessary to decompress all the way to the maxillary strut for complete decompression, because the oculomotor nerve gradually approaches the optic strut rather than the lower middle part of the superior orbital fissure when anteriorly and superiorly traveling outside the cavernous sinus. The decompression ranges anteroposteriorly from the orbital apex to the lower angle of the optic strut triangle, which corresponds to the proximal ring of the paraclinoid ICA. The order of decompression of the superior and inferior walls of the optic canal is also highly important (Figure 1B). Decompression of the inferomedial wall of the optic canal was performed first to determine the specific location and morphology of the optic nerve, as well as the drilling range of the superior and inferior walls. The inferior wall was ground last because of the risk of oozing blood from the intercavernous sinus, which may impede the surgical process. During the treatment of the superior wall of the optic nerve, the surgeon must be prepared for encountering the risk of cerebrospinal fluid leakage resulting from the anterior skin base dura mater rupture and laceration.

The internal surface of the sphenoid sinus corresponds to the LOCR on the lateral wall of the sphenoid sinus. As recognized by the regional anatomical study of dried skull and cadaveric head, the oculomotor nerve separates into superior and inferior branches before entering the superior orbital fissure along the inferior lateral margin of the optic strut. The oculomotor nerve closely adjacent to the lateral edge of the optic strut could also be clearly observed under the endoscope (Figures 2A–D), which is theoretically the narrowest point of the oculomotor nerve in the fissure segment. It is, therefore, plausible to suggest that optic strut drilling is necessary to achieve a satisfactory decompression of the oculomotor nerve fissure segment. At present, decompression surgeries of the superior orbital fissure, orbital apex and optic canal are also performed in some cases of traumatic oculomotor nerve palsy, achieving good results (10, 29), but none of them involve optic strut drilling. It was found that 270° decompression could be achieved after the optic strut was ground (Figure 1A), and the medial margin of the oculomotor nerve fissure segment could be sufficiently decompressed after the orbital apex and superior orbital fissure were ground in turn. In Case 2, the conservative treatment had a poor effect during the first 3 months, but the extraocular muscles showed signs of recovery immediately after optic strut drilling. It can be seen that the combination with optic strut drilling may be more effective than the decompression of superior orbital fissure and orbital apex alone, but multicenter clinical trials are needed for further verification. In addition, optic canal decompression is not necessary for patients with pure traumatic oculomotor nerve palsy if the sphenoid sinus pneumatizes into the optic strut, the space of the optic strut triangle is large enough, and the optic strut can be directly ground by an emery drill in an eggshelled manner. In the case of poor optic strut pneumatization, however, optic canal decompression is necessary, by which the optic nerve can be slightly displaced upward to be protected at the time of optic strut drilling, and the optic strut triangle can be more clearly delineated to guide the surgery. On the above basis, a protocol for the decompression surgery of the orbital apex, superior orbital fissure, optic canal and optic strut in turn was developed.

As for the surgical plan of decompression of the superior orbital fissure, Abuzayed et al. discussed Optic Canal Decompression and medial orbitotomy as well as emphasized the feasibility of transnasal endoscopic access to the orbital apex and medial wall of the orbita. they compared different approaches to intraorbital Space,the advantages of endoscopic transnasal approach, such as excellent visual field, minimal invasion, and no traction of brain and nerve, have been discussed (30). Meanwhile, During clinical practice, we found that transcranial approach is suitable for lesions which on the lateral side of the superior orbital fissure,It can also decompress the oculomotor nerve. However, craniotomy is invasive and requires brain retraction, and it cannot effectively decompress the inferior medial side of the optic strut in the orbital apex. The transnasal transsphenoidal approach can reach the medial and inferior of the superior orbital fissure, but it lacks a direct visual to the orbital apex, The freedom of surgery is also somewhat worse when dealing with the lateral of LOCR. Transorbital approach can reach the lateral, medial and superior sides of the orbit, but it requires traction and compression of the orbital structure, which increases the risk of secondary injury to the orbital nerve, and may damage the eyeball support system, leading to deformity or ocular muscle dysfunction.

Finally, it is necessary to rule out oculomotor nerve injuries other than those in the oculomotor nerve fissure segment before surgery for patients diagnosed with traumatic oculomotor nerve palsy, so high-resolution MRI is further recommended, especially when no fracture is found (31–33). Possible vascular lesions such as pseudoaneurysm, aneurysm or carotid-cavernous sinus fistula must be excluded by cranial CT, and 3D skull reconstruction should be further performed to evaluate the site of superior orbital fissure fracture, the degree of sphenoid sinus pneumatization and the location of ICA, and to determine whether neuronavigation and pre-surgical planning are required.

## Conclusion

The optic strut triangle is an important anatomical landmark for oculomotor nerve decompression, and optic strut drilling is necessary for oculomotor nerve decompression. Through the sequential transnasal endoscopic decompression of the orbital apex, the superior orbital fissure, the optic canal and the optic strut, the oculomotor nerve fracture segment in traumatic oculomotor nerve palsy patients with fractures of the medial wall of the superior orbital fissure can be safely and effectively decompressed leading to help forrecovery of neurological function. This study aims to provide a new surgical plan for traumatic oculomotor nerve palsy, and a scheme for surgical treatment of orbital apex syndrome and SOFS.

## Data Availability

The raw data supporting the conclusions of this article will be made available by the authors, without undue reservation.
